# A Community Benchmark for the Automated Segmentation of Pediatric Neuroblastoma on Multi-Modal MRI: Design and Results of the SPPIN Challenge at MICCAI 2023

**DOI:** 10.3390/bioengineering12111157

**Published:** 2025-10-26

**Authors:** Myrthe A. D. Buser, Dominique C. Simons, Matthijs Fitski, Marc H. W. A. Wijnen, Annemieke S. Littooij, Annemiek H. ter Brugge, Iris N. Vos, Markus H. A. Janse, Mathijs de Boer, Rens ter Maat, Junya Sato, Shoji Kido, Satoshi Kondo, Satoshi Kasai, Marek Wodzinski, Henning Müller, Jin Ye, Junjun He, Yannick Kirchhoff, Maximilian R. Rokkus, Gao Haokai, Matías Fernández-Patón, Diana Veiga-Canuto, David G. Ellis, Michele Aizenberg, Bas H. M. van der Velden, Hugo Kuijf, Alberto de Luca, Alida F. W. van der Steeg

**Affiliations:** 1 Princess Máxima Center for Pediatric Oncology, 3584 CS Utrecht, The Netherlands; m.a.d.buser-3@prinsesmaximacentrum.nl (M.A.D.B.);; 2 Department of Radiology, University Medical Center Utrecht, 3584 EA Utrecht, The Netherlands; 3 Image Sciences Institute, University Medical Center Utrecht, Utrecht University, 3508 GA Utrecht, The Netherlands; 4 Department of Artificial Intelligence in Diagnostic Radiology, Osaka University Graduate School of Medicine, Osaka 545-8585, Japan; 5 Department of Sciences and Informatics, Muroran Institute of Technology, Hokkaido 050-8585, Japan; 6 Department of Intelligent Information Engineering, Fujita Health University, Aichi 470-1192, Japan; 7 Department of Measurement and Electronics, University of Krakow, 30-059 Krakow, Poland; 8 Institute of Informatics, HES-SO, 3960 Sierre, Switzerland; 9 Department of Radiology and Informatics, University of Geneva, 1211 Geneva, Switzerland; 10 Shanghai Artificial Intelligence Laboratory, Shanghai 200232, China; 11 Cancer Research Center, 69120 Heidelberg, Germany; 12 School of Computer Science, South China Normal University, Guangzhou 510631, China; 13 La Fe Health Research Institute, 46026 Valencia, Spain; 14 University of Nebraska Medical Center, Omaha, NE 68198, USA; 15 Wageningen Food Safety Research, 6708 WB Wageningen, The Netherlands

**Keywords:** neuroblastoma, MRI, segmentation, 3D visualization, challenge

## Abstract

Surgery plays a key role in treating neuroblastoma. To assist surgical planning, anatomical 3D models derived from the segmentation of anatomical structures on MRI scans are often used. Automation using deep learning can make segmentations less time-consuming and more reliable. We organized the Surgical Planning in PedIatric Neuroblastoma (SPPIN) challenge, to stimulate developments and benchmarking of automatic segmentation of neuroblastoma on MRI. SPPIN is the first segmentation challenge in extracranial pediatric oncology. Nine teams provided a valid submission. Evaluation was based on the Dice similarity coefficient (Dice score), the 95th percentile of the Hausdorff distance (HD95), and the volumetric similarity (VS). A combination of these scores determined the ranking of the teams. The spread in the median evaluation scores per team was large (Dice: 0.21–0.82; HD95: 63.31–7.69; VS: 0.31–0.91). The top-performing team achieved a median Dice score of 0.82 (with an HD95 of 7.69 mm and a VS of 0.91) using a large, pre-trained model. However, in the pre-operative segmentations, significantly lower evaluation scores were observed. Our results indicate that pre-training might be useful in small, pediatric datasets. Although the general results of the winning team were high, they were insufficient to use for surgical planning in small, pre-operative tumors.

## 1. Introduction

Neuroblastoma is one of the most common extracranial solid tumors in children [1]. The worldwide incidence of neuroblastoma is about 11 per million children under the age of 15 [2]. Neuroblastoma accounts for 15% of cancer-related deaths in children [1,3]. Treatment includes chemotherapy, immunotherapy, surgery, and radiotherapy. While the specific treatment strategy depends on multiple factors such as age, tumor biology, and image-defined risk factors, chemotherapy followed by surgery is one of the mainstays of treatment [1,4]. Surgery is used to gain local control, aiming to debulk at least 95% of tumor tissue [5]. The majority of neuroblastomas are located in the abdomen where surgical debulking can be challenging, due to adherence to important abdominal structures, such as the spleen, liver, kidneys, and ureter, and abdominal vessels such as the aorta, vena cava, and renal vessels in the vicinity of the tumor [5,6].

To safely remove as much tumor tissue as possible, it is essential for the surgeon to understand the position of the neuroblastoma in relation to important abdominal structures. Currently, pre-operative imaging such as magnetic resonance imaging (MRI) is used to investigate the anatomical situation of the patient. In other pediatric tumors, creating 3D models of the tumor in relation with other important structures from the MRI images was found to increase the anatomical understanding of the surgeon during surgical planning, potentially leading to faster procedures with better surgical outcomes [7,8,9]. However, creating 3D models of neuroblastoma and important abdominal structures currently relies on manual or semi-automatic segmentation (e.g., delineation), which is user-dependent and a time-consuming process [10,11].

A promising way to automate the segmentation process is by using deep learning (DL) [12]. DL has been applied in segmentation of pediatric oncology on MRI, for example, in Wilms tumor, osteosarcoma, and retinoblastoma [13,14,15,16]. In general, automating the segmentation of neuroblastoma is not straightforward because its rarity inherently leads to limited sample sizes. Furthermore, the location, size, shape, and image characteristics of the tumor can vary greatly between patients due to the heterogeneous biology of neuroblastoma [17]. Lastly, chemotherapy-induced changes often decrease the size of the tumor and visibility of the tumor boundaries, further complicating its segmentation after treatment. To date, only two studies have focused on segmentation of neuroblastoma using deep learning [10,11]. The method described in those two papers was able to segment tumors on diagnostic MRI with high accuracy. However, it remains unclear whether this can be generalized to pre-operative planning using MRI scans of pre-treated tumors.

To address this gap of knowledge, we organized a community challenge focusing on fully automatic neuroblastoma segmentation. In the past, similar challenges, such as BraTS (for brain tumor segmentation, including pediatric data) or LiTS (for adult liver tumor segmentation), have led to significant improvements in algorithm development and clinical applicability. The Surgical Planning in PedIatric Neuroblastoma (SPPIN) challenge was hosted online in 2023, with a concluding, in-person challenge session in conjunction with the 26th International Conference on Medical Image Computing and Computer Assisted Intervention (MICCAI) in Vancouver, Canada. Our challenge focused on fully automated neuroblastoma segmentation using multi-modal MRI scans during multiple stages in the treatment process, with a focus on accurate segmentation of post-chemotherapy pre-operative scans [18]. The addition of both chemo-naïve and post-chemotherapy scans in the dataset increased the dataset size and provided the possibility of comparing the segmentation results in both groups. The aim of this paper is to describe the challenge set-up, explain the methods of the participating teams, and provide an objective assessment of the performance of the methods with an eye on their potential future translation to support surgical planning. The main contribution of this paper is providing a research benchmark for pediatric neuroblastoma segmentation, by utilizing a community challenge to provide an overview of the current segmentation strategies. The secondary contribution is the special focus on surgical planning, as this is the first paper investigating the difference in segmentation outcomes in chemo-naïve, diagnostic, and post-chemotherapy, pre-operative imaging.

## 2. Materials and Methods

### 2.1. Challenge Design

The SPPIN challenge was organized by the Princess Máxima Center for Pediatric Oncology. The challenge was organized in conjunction with MICCAI 2023, and in line with their policy; the challenge design was peer-reviewed and published before the start of the challenge [18]. SPPIN was also hosted online at Grand Challenge, with an in-person closing session hosted as a satellite event of MICCAI 2023 on 8 October 2023. Teams with members of the organizers’ institute were not allowed to participate. Detailed information about the challenge is available at https://sppin.grand-challenge.org/ (accessed on 1 September 2025). Only fully automated methods were allowed. Use of external data to (pre-)train or fine-tune the method was only allowed when a publicly available dataset was used and cited.

The challenge consisted of three phases, each using separate parts of the challenge data (see Section 2.2).

1. The training phase ran from 14 April 2023 to 1 September 2023, for which data was available after signing a data release form.
2. The preliminary test phase ran from 1 May 2023 to 1 September 2023. In the preliminary test phase, teams could test their method to ensure that the method behaved as expected. Teams had 5 attempts to submit their method in the preliminary test phase, and the best scoring method compared to the previous attempts was directly posted on the live leaderboard. This phase allowed teams to test and debug their methods and prevented the tweaking of results for the final leaderboard.
3. The final test phase ran from the 14 August 2023 to 1 September 2023. Teams only had one attempt, and the results of this leaderboard were hidden until the SPPIN challenge session during MICCAI on 8 October 2023.

To participate in a phase, teams submitted their automated segmentation methods as a docker container on Grand Challenge, for which instructions were available [19]. After uploading their method, the segmentation and evaluation were performed automatically within the platform. As per the restrictions of Grand Challenge, the computation time for one scan was limited to 20 min.

### 2.2. Datasets

**Figure 1:**
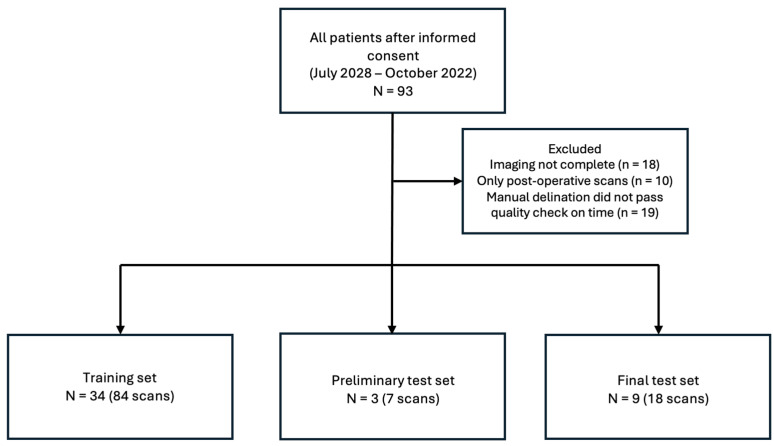
Overview of patient inclusion. The training set refers to the set of scans the teams received at the beginning of the challenge. The preliminary test set was used as check for the teams, with results being posted directly on the live leaderboard. The final test set was used to determine the final leaderboard and winners of the SPPIN challenge.

We retrospectively included data of 93 neuroblastoma patients aged 0–18 treated in the Princess Máxima Center for Pediatric Oncology during the period of July 2018 to October 2022. The study was conducted in accordance with the Declaration of Helsinki. For all patients, informed consent was present, and all patients received treatment according to the Dutch Childhood Oncology Group (DCOG) NBL 2009 protocol [20]. Patients were excluded from the dataset when the imaging sequences were not complete (*n* = 18), only post-operative scans were present (*n* = 10), or if the manual delineation (Section 2.2.2) did not pass the quality check due to time constraints while organizing the challenge (*n* = 19). The final test included data of 9 patients, totaling 18 scans. An overview of patient inclusion can be seen in Figure 1. Clinical characteristics were collected for analysis.

#### 2.2.1. Magnetic Resonance Imaging

**Figure 2:**
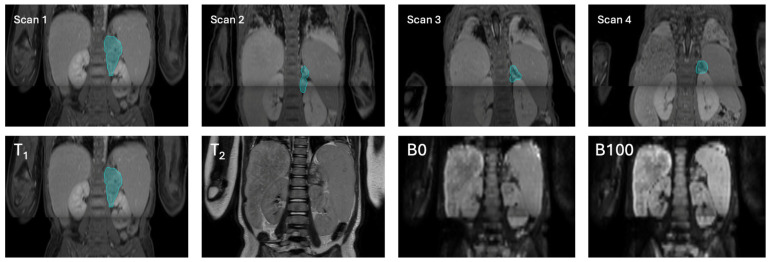
Example of multiple scans belonging to one patient. The top row depicts several scanning moments throughout the treatment process, with scan 1 being the diagnostic scan, scans 2 and 3 performed during chemotherapy at different time moments, and scan 4 being the pre-operative scan at the end of the chemotherapy. The bottom row shows the four MRI scans belonging to the diagnostic scans. From left to right: the T_1_-weighted contrast-enhanced scan, the T_2_-weighted scan, the DWI scan (b = 0 s/mm^2^), and the DWI scan (b = 100 s/mm^2^). The ground truth is depicted in blue on the T_1_-weighted contrast-enhanced scan, where the ground truth was created. The line present in most images is the result of the combination of multiple scanning fields of view as present in the used protocol.

MRI scanning was performed on a 1.5T unit (Ingenia; Philips Medical Systems, Best, The Netherlands). The imaging included fat-suppressed T_1_-weighted images with and without intravenous contrast (Gadovist, Bayer Pharma, Berlin, Germany, 0.1 mmol/kg body weight), a 3D T_2_-weighted image, and diffusion-weighted images (DWI) (Table 1). Scans were pseudonymized and exported to NIFTI. No further preprocessing or registration was conducted, to simulate the initial clinical situation. An example of different sequences included per patient is shown in Figure 2.

All scanning moments before surgery were included in the dataset for the included patients. Although the focus of the challenge was pre-operative segmentation of pre-treated tumors, the inclusion of chemo-naïve diagnostic scans increased the overall dataset size and enabled direct comparison of these groups to investigate the effect of chemotherapy on the segmentation results. For each patient, the scanning dates of their scans were provided, but no additional (clinical) information was included.

#### 2.2.2. Dataset Splits

The included patients were split into train (*n* = 34 patients with 78 scans), preliminary test (*n* = 3 patients with 7 scans), and final test sets (*n* = 11 patients with 24 scans). After the end of the challenge, 2 patients were removed from the final test set due to clinical inconsistencies, leading to a final test set including 9 patients and 18 scans (see Section 2.2.3). The reported numbers are consistent with the final analysis. Baseline characteristics are displayed in Table 2. The SPPIN training data will remain available upon request with the corresponding author.

#### 2.2.3. Manual Annotation

Scans were manually annotated by delineating the contour of neuroblastoma using a custom tool created in MeVisLab (version 3.4.2) [21]. Firstly, five trained technical medicine students performed the delineation under direct supervision of two experienced technical physicians. Each rater annotated a non-overlapping subset of the dataset. The delineation protocol was approved by a dedicated pediatric radiologist (Supplementary File A). For the final test set, the segmentations were checked and, if needed, adjusted by two experienced technical physicians. Lastly, after the challenge submissions closed, a pediatric radiologist performed a systematic check of the annotations in the final test set and, based on this, two patients were removed from analysis.

### 2.3. Challenge Evaluation

#### 2.3.1. Automated Evaluation and Ranking

For each submission, individual rankings (1–9) were assigned based on the Dice similarity coefficient (Dice score), the 95th percentile Hausdorff distance (HD95), and the volumetric similarity (VS) [22]. The overall ranking was obtained by averaging the three individual ranks, with the resulting value rounded up to the nearest integer. In cases where submissions achieved identical final scores, the Dice score ranking was used as a tiebreaker, given its prominence as the standard metric for segmentation performance. Due to the nature of the challenge design, no missing results were present. Empty segmentations were given an HD95 of infinity (in the statistics depicted as NaN). Evaluation and statistical analysis were performed in Python 3.8. The Python code used for the automated evaluation of the segmentations can be referenced at the challenge Github page [19].

#### 2.3.2. Confounder Analysis

We evaluated whether two key clinical factors, namely, tumor size and treatment, influenced segmentation performance across teams. To this end, we applied the Kruskal–Wallis test to assess whether there were statistically significant differences in segmentation outcomes between diagnostic and post-chemotherapy scans for each team. In addition, we generated scatterplots of Dice score versus tumor size for each team to visually examine potential associations between segmentation performance and tumor size.

## 3. Results

### 3.1. Challenge Submission and Participating Teams

For the preliminary test phase, 11 teams submitted 24 valid segmentation methods. For the final test phase, 12 teams submitted a single segmentation method each, resulting in 12 valid methods. This paper describes the results of the submission of nine teams that submitted both a valid method in the final test phase and provided a complete description of the used method. The key characteristics of the teams are reported below; a complete overview of their methods can be seen in Supplementary File B. The technical details are summarized in Table 3.

*Blackbean:* The winning team used a large network (STU-Net), pre-trained on a large dataset of CT scans including labels for > anatomical structures [23,24,25].

*Jishenyu:* This team used a strategy-based nnU-Net [24], using all input sequences after registration to the T_1_-weighted image.

*Ouradiology*: An ensemble of multiple nnU-Net [24] models, based on 5-fold cross validation, was used by this team.

*Drehimpuls*: This team used a combination of an nnU-Net [24] trained on all data with a ‘fallback’ network which was trained on all segmentations obtaining a Dice of >0.5 during 5-fold cross validation. Although this team tested several variants of the basic nnU-Net, they found that this did not enhance performance.

*SK*: By using five consecutive slices in the transverse plane, this team created a 2.5D segmentation method, using T_1_-weighted and T_2_-weighted images.

*AGHSSO:* Heavy data augmentation, using all four sequences, was the key to this team’s segmentation method.

*UNMC*: This team used all four input sequences and performed background cropping and data augmentation before training their segmentation method.

*SPPIN_SCNU:* This team used T_1_-weighted images to train a U-Net [24] with a transformer as encoder.

*GIBI230:* This team used a previously developed segmentation method with T_2_-images as input.

### 3.2. Metric Values and Ranking

**Figure 3:**
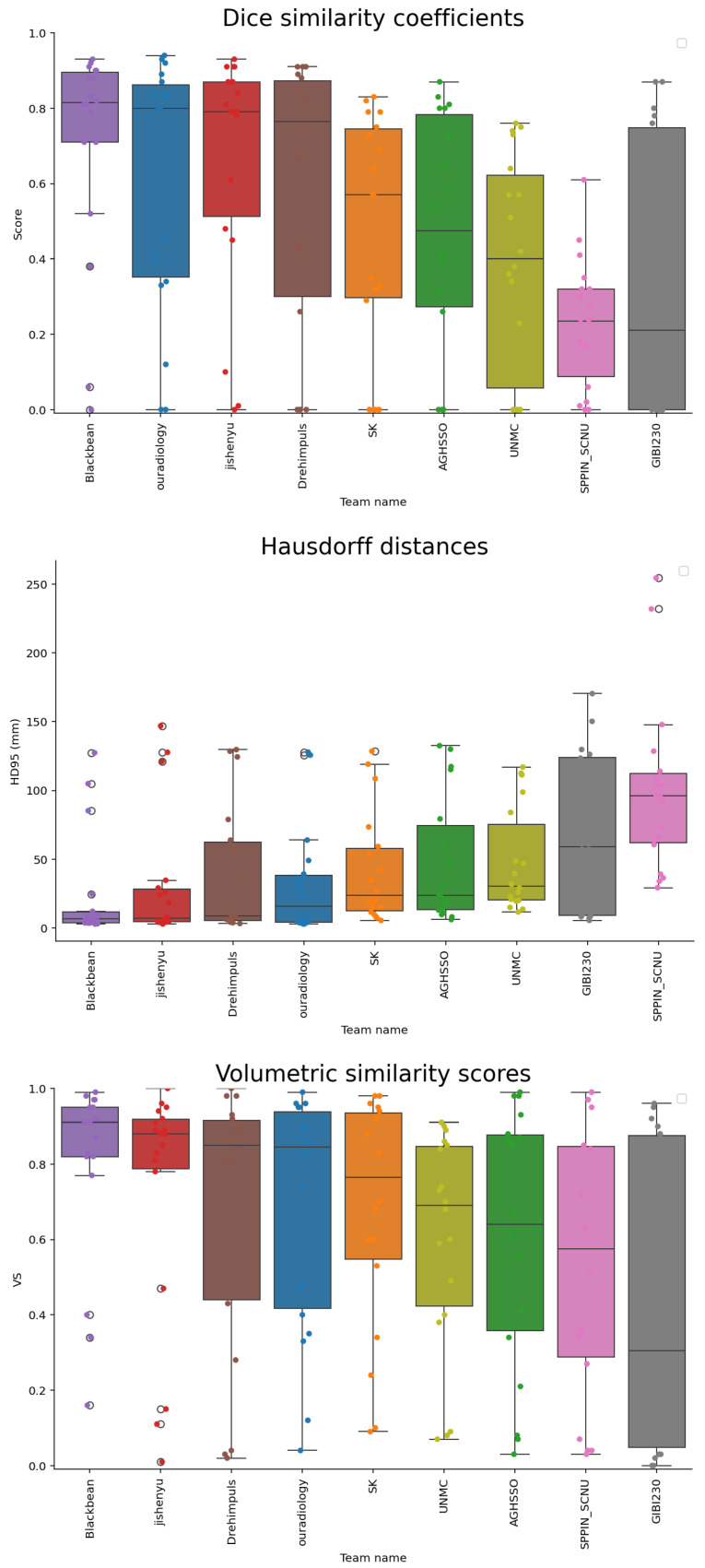
The three evaluation parameters for each team, in order of the individual evaluation metric. Plotted as median (line), IQR 1 and 3 (whiskers) and outliers (circles). The colors indicate the different teams and the points each individual score. From top to bottom: the Dice similarity coefficients (higher is better); the 95th percentile of the Hausdorff distances (HD95) (lower is better; please note that HD95 values resulting from an empty segmentation are ignored while plotting); the volumetric similarity scores (higher is better).

**Figure 4:**
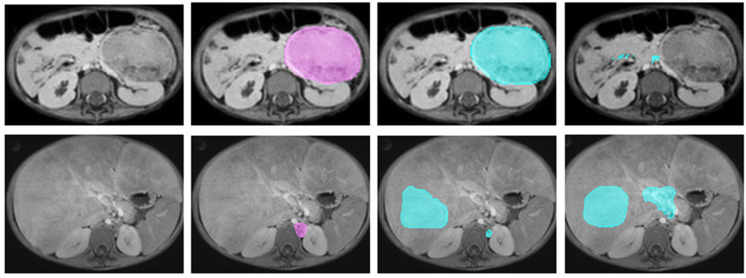
Overview of the segmentations of the patients belonging to the segmentation with the highest single Dice score (top row) and lowest non-zero Dice score (bottom row). The ground truth is depicted in pink, the teams’ output in blue. The top row: Test patient 5_1 (diagnostic). From left to right: T_1_-weighted scan, ground truth in pink, highest scoring: team ouradiology (Dice = 0.93), lowest scoring: team SPPIN_SCNU (Dice = 0.32). This tumor is clearly defined and located in the left peritoneal space. Bottom row: Test patient 3_3 (post-chemo). From left to right: T_1_-weighted scan, ground truth, highest scoring: team Blackbean (Dice = 0.06), lowest scoring: team jishenyu (Dice = 0.01). This small and not well-defined primary tumor is accompanied by diffusely infiltrated liver metastases.

In Table 4, the metrics for each team are presented. The highest scoring team had a median Dice score of 0.82, a median HD95 of 7.69 mm, and a median VS of 0.91. The lowest ranked team achieved a median Dice score of 0.21, a median HD95 of 63.41 mm, and a VS of 0.31. The distribution of these scores per team can be seen in Figure 3. In Figure 4, an overview of the tumors for which the best and worst Dice scores were observed, is provided. The tumor for which the highest Dice score was observed is a large, clearly defined tumor in a chemo-naïve patient. The tumor for which the lowest Dice score was observed was small and not defined, in a pre-treated patient. Noticeable are the diffuse liver metastases, incorrectly segmented as tumor by all teams.

### 3.3. Confounders

The median Dice scores for the three top scoring teams and the lowest scoring team were significantly different between the diagnostic (chemo-naïve) and post-chemotherapy scans. For the winning team, the median score for diagnostic scans was 0.81, in contrast to post-chemotherapy scans with a median Dice score of 0.47 (*p* = 0.01). A significant difference for HD95 was only observed in one team, where only SPPIN_SNCU had a lower HD95 for the diagnostic scans. For the volumetric similarity, only team Jishenyu had a significant difference between the diagnostic and post-chemotherapy segmentations. An overview of the complete analysis can be seen in Table 5.

**Figure 5:**
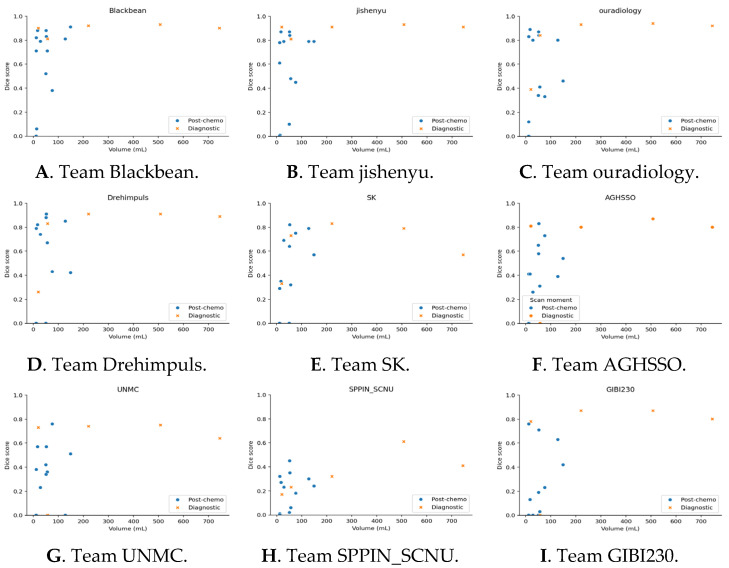
The Dice scores plotted against the tumor volume in mL for each team. Orange crosses depict a tumor segmentation of a diagnostic MRI scan, blue points that of a post-chemotherapy scan.

The effect of tumor size and pre-treatment is depicted in Figure 5. No clear effect of tumor size on the Dice score can be observed for each team.

## 4. Discussion

Surgical Planning in PedIatric Neuroblastoma is the first medical imaging challenge in the field of extracranial pediatric oncology. In total, nine teams were eligible for the final leaderboard. The scores of the participating teams varied widely, with the highest ranking submission achieving a median Dice score of 0.82, a median HD95 of 7.69 mm, and a VS of 0.91, while the lowest ranking submission had a median Dice score of 0.21, a median HD95 of 63.31 mm, and a VS of 0.31. A significant difference between the Dice score in chemo-naïve and pre-treated tumors was observed for the top three teams. The segmentation of the pre-operative scans was the primary focus of the SPPIN challenge, but despite the high overall results, these segmentation results showed room for improvement.

Most submissions, including the top three, used a variation of nnU-Net as a base architecture [24]. While this is perhaps unsurprising, as nnU-Net has shown excellent performance on most leaderboards of recent challenges [24], our results highlight its potential also in relatively small datasets with heterogeneous tumors. Other teams, such as SK, AGHSSO, and UNMC, used methods other than nnU-Net, but these methods did not achieve the same level of performance. The highest ranking team (Blackbean, Shanghai Artificial Intelligence Laboratory, Shanghai, China) used a Scalable and Transferable nnU-Net [23] as segmentation method. The unique addition of Blackbean was its extensive pre-training on a large (>1000 scans) dataset of CT-scans labeled with anatomical structures. This suggests that pre-training is valuable for segmentation in small, heterogenous datasets such as ours, even across modalities [26]. Together, it appears that major sources of improvement for individual challenges likely lie in data curation, pre-training, and preprocessing.

Another important part of the methods of the teams to consider is the input sequence. The majority of the teams used a single input method. As the ground truth was created on the T1-weighted contrast-enhanced imaging, most teams used this as input. This was partly expected, as the neuroblastoma has the highest visibility on this sequence; this is also the reason why the ground truth labels were annotated on this sequence. Among the submissions that did use sequences besides the T1-weighted scan, there was a large variability in the preprocessing steps performed. For example, some teams chose to just perform a resampling of the images, whereas other teams performed a co-registration of the scans. This makes comparing the added value of several sequences difficult. One team used T2-weighted imaging as the sole input (GIBI230). Although the method of this team showed good performance in previous publications, with Dice scores > 0.90, they ranked last in our challenge [10,11]. However, as they used their T_2_-based method on T_1_-weighted scans, it is difficult to draw a general conclusion about the usefulness of T_2_-based segmentation in neuroblastoma. This also holds true when looking at the other teams that used T2-weighted imaging as additional input, with (*n* = 4) or without the addition of DWIs (*n* = 1). For the DWI input scans, we selected the scans with a B-value of 0 and 100 as these scans better preserve anatomical detail compared to higher B-values. Nevertheless, the increased diffusion signal at higher B-values, often observed in tumors, could assist in segmenting tumors that are otherwise difficult to delineate. However, even if T1-based segmentation alone is enough to provide the anatomical information needed for pre-operative planning, other sequences can be used to add additional (functional) information [27]. In conclusion, due to the diversity in the methods used and the subsequent segmentation results, it was not possible to derive a conclusion on the best input sequences for neuroblastoma segmentation. Further challenges may consider providing pre-registered data to stimulate multi-sequence input methods.

The highest ranking team scored a median Dice score of 0.82, which is lower than the best performing methods in other oncological segmentation challenges [10,11]. When looking at the study of Veiga-Canuto et al. on neuroblastoma segmentation, it is noticeable that their reported median Dice score of >0.9 is significantly higher than the median Dice score of our highest ranking team [10,11]. However, Veiga-Canuto only used chemo-naïve, diagnostic MRI scans in this study. Indeed, when looking at the median Dice score in only diagnostic tumors for the highest scoring team (Dice = 0.89), this is in line with the reported scores of Veiga-Canuto. The top three scoring teams all showed a statistically significant difference in Dice score between diagnostic and post-chemotherapy scans, further supporting the inherent difficulty of segmentation in pre-treated tumors. Although diagnostic scans might seem less relevant for the aim of pre-operative planning, they carry important information about the extent and location of the primary disease, and they provide a valuable enlargement of our limited samples size. Moreover, diagnostic MRI scans do have value in clinical planning, for example, in the localization of small lesions after chemotherapy. Nevertheless, the addition of chemo-naïve patients positively influenced the final results of the challenge, obscuring the fact that several tumors scored low even in high-performing teams.

There are several potential strategies to improve the segmentation results specifically in pre-operative imaging. One team aimed to improve the segmentation of small tumors by implementing a specifically designed fallback network. Despite this, their final results still included six tumors with a Dice score of <0.5. Other strategies that remain untested in our challenge include longitudinal segmentation where diagnostic scans are used to inform the segmentation for the pre-operative scans [28] and the addition of attention-based networks to capture long-range patterns in the data [29]. Data-specific approaches could include pre-operative imaging-specific data augmentation, potentially using generative models [30], or federated learning can be a strategy to increase the dataset size without the need for data sharing between centers [31,32].

The literature on deep learning-based segmentation in pediatric imaging remains limited, especially in extracranial oncology. Besides the articles by Veiga-Canuto, no prior studies have investigated automated MRI-based segmentation of neuroblastoma [10,11]. While some studies have explored deep learning for Wilms tumor segmentation, one of the most common abdominal tumors in children, methodological differences, as well as the distinct growth patterns and imaging characteristics of Wilms tumors, constrain direct comparison with our findings [14,15,33]. Moreover, the only publicly available pediatric segmentation challenge, BraTS, focuses on brain tumors, which differ substantially from abdominal tumors [34].

Our challenge had several limitations. First, our challenge included a limited number of patients, reflecting the rarity of neuroblastoma. While the small training dataset may have limited the overall segmentation performance and the small, single-center test set constrains conclusions about generalizability, the findings are valuable as an initial benchmark for automated segmentation in extracranial pediatric oncology. Despite the limited sample size, the performance trends across participating teams provides a meaningful starting point for future, larger-scale studies for neuroblastoma segmentation. Moreover, a significant number of segmentations (*n* = 19) failed to pass the quality check, which led to a smaller dataset than initially planned and potential bias for the exclusion of harder-to-interpret tumors. As these segmentations were pre-operative scans only, this can potentially lead to an overestimation of the performance in that group only. Therefore, while potentially overestimating the overall performance, it does not change our conclusion about the inherent difficulty of segmentation in pre-treated neuroblastoma. Next, there was a significant difference in tumor volume between the train and final test set on the one hand and the preliminary test set on the other hand. However, the effect of this on the final results was most likely limited as the preliminary test set was only used by the teams to check if their method was feasible. Furthermore, interobserver variability could not be determined as there was no overlap between annotations per observer. However, extensive quality control was present for the test set to ensure the evaluation was based on high quality segmentations. As this was not in place for the training set, this could have had a negative impact on the overall challenge results. Lastly, as observer variability was not determined, the limited performance in some of the tumors could not be placed in context. Another limitation was the way of dealing with the location of the arteries and veins within segmentation of the neuroblastoma. To ensure reproducibility, our segmentation tool was only able to create closed contours, but this posed a challenge for the segmentation of the tumor in proximity to (big) vessels. Practically, only vessels close to the border of the neuroblastoma could be excluded from the tumor segmentation, whereas vessels fully encased by the tumor were included in the ground truth segmentations. This resulted in small but consistent errors in the ground truth tumor segmentations. However, as (manual) vessel segmentation is the next step in the workflow of creating the 3D models for neuroblastoma, this can be simply dealt with in the post-processing steps [27]. We used well established evaluation parameters for our challenge, but this might not reflect clinical applicability completely. Further research is needed to address clinical applicability of the developed 3D models, in addition to image analysis evaluation parameters. Currently, the segmentation algorithms focus solely on neuroblastoma segmentation as first proof of concept. However, for a clinically applicable model, the important vessels (including but not limited to the aorta, superior mesenteric artery, inferior mesenteric artery, renal arteries, vena cava, renal veins, and portal veins) and organs of interest (including the kidneys, liver, spleen, and pancreas) need to be included in the segmentation model.

## 5. Conclusions

The SPPIN challenge, aimed at providing a research benchmark for neuroblastoma segmentation, concluded that current deep learning methods can achieve good results for the segmentation of tumors before treatment (Dice > 0.8), but also that the automated segmentation of smaller, pre-treated tumors, is lacking (Dice < 0.50). The limited performance in pre-treated tumors reflects a current inability to use automated segmentation methods in surgical planning, the overall aim of our challenge. Pre-training of segmentation methods seems promising to support the training of automated segmentation methods in small, heterogenous datasets such as those supplied during this challenge. To create clinically applicable 3D models, more reliable and extensive segmentation models are needed with a focus on small, post-chemotherapy tumors and the inclusion of other anatomical structures.

## Figures and Tables

**Table 1:** Magnetic resonance imaging scanning parameters. The Z dimension (feet-head direction) of the scans is variable.

Sequence	T_1_-Weighted Gradient Echo	T_2_-Weighted Spin Echo	Diffusion-Weighted Imaging
**Repetition time (ms)**	6.1	458	2537
**Echo time (ms)**	2.9	90	76.4
**Voxel size (mm** ^3^ **)**	0.71 × 0.71 × 3	0.833 × 0.833 × 1.15	1.39 × 1.39 × 5
**Dimensions (voxels)**	560 × 560 × Z	480 × 480 × Z	288 × 288 × Z
**b-values (s/mm^2^)**	N/A	N/A	0, 100
**Contrast**	Gadovist, 0.1 mmol/kg body weight	-	-

**Table 2:** Baseline characteristics of the three challenge datasets. The datasets were split on a patient level.

	Training Set (*n* = 34)	Preliminary Test Set (*n* = 3)	Final Test Set (*n* = 9)
**Number of included scans**	84	7	18
**Age in years at diagnosis (median, min–max)**	2.5 (0–11)	2 (0–3)	2 (0–12)
**Sex**	Male = 17	Male = 2	Male = 6
	Female = 17	Female = 1	Female = 3
**Tumor volume in mL (median, min–max)**	53.48 (2.03–1249.7)	7.7 (4.28–304.7)	51.1 (4.9–745.4)

**Table 3:** Overview of the segmentation method used by each team, from the highest to the lowest ranking team. If an entry was reported but not applied, this was denoted by ‘-’. If an entry was not reported, it was denoted as Not Reported (N.R.). T_1_ = T_1_-weighted contrast-enhanced scan; T_2_ = T_2_-weighted scan; DWI_b0 = diffusion-weighted image, b-value 0; DWI_b100 = diffusion-weighted image, b-value 100.

Team	Network	Input	Pre-Training	Preprocessing	Patch Size	Data Augmentation	Loss	Post-Processing
**Blackbean**	Scalable and Transferable U-Net	T_1_	TotalSegmentor dataset	nnU-Net default	Yes, size not specified	Yes	Dice loss	N.R.
**jishenyu**	nnU-Net	T_1_, T_2_, DWI_b0, DWI_b100	Pre-trained nnU-Net weights	Registration of input to T_1_	N.R.	Yes	Dice + cross entropy loss	N.R.
**Ouradiology**	nnU-Net	T_1_	N.R.	nnU-Net default	64 × 288 × 288	N.R.	nnU-Net default	nnU-Net default
**Drehimpuls**	nnU-Net	T_1_, T_2_, DWI_b0, DWI_b100	N.R.	Resampling input to T_1_ space, z-score normalization	128 × 128 × 128	N.R.	Fbeta + cross entropy loss	Fallback network
**SK**	2.5D U-Net, with EfficientNet as encoder	T_1_, T_2_	N.R.	Resampling input to T_1_ space	N.R.	-	Dice + cross entropy loss	N.R.
**AGHSSO**	ResUNet	T_1_, T_2_, DWI_b0, DWI_b100	N.R.	Resampling input to [224^3^], normalization	Whole image	Yes	Soft + focal loss	Delete connected components < 20 voxels
**UNMC**	DynUNet	T_1_, T_2_, DWI_b0, DWI_b100	N.R.	Registration of input T_1_, foreground cropping, z-score intensity normalization, linear resampling [192 × 192 × 192]	N.R.	Yes	Dice loss	Selection of largest component
**SPPIN_SCNU**	UNETR	T_1_	N.R.	N.R.	N.R.	Yes	N.R.	N.R.
**GIBI230**	nnU-Net	T_2_	N.R.	Resampling to [0.695 × 0.695 × 8] mm voxel size, z-score normalization	N.R.	N.R.	Dice loss	N.R.

**Table 4:** The scores and rankings of the participating teams. Dice = Dice similarity coefficient. HD95 = the 95th percentile of the Hausdorff distance. VS = volumetric similarity.

Team Name	Median Dice [Min–Max]	Ranking Dice	Median HD95 (mm)	Ranking HD95	Median VS	Ranking VS	Final Ranking
**Blackbean**	0.82 [0.00–0.93]	1	7.69 [2.82–127.35]	1	0.91 [0.16–0.99]	1	1
**Jishenyu**	0.79 [0.00–0.93]	3	13.19 [2.83–146.91]	2	0.86 [0.01–1.00]	2	2
**Ouradiology**	0.80 [0.00– 0.94]	2	15.91 [2.83–127.82]	3	0.85 [0.04–0.99]	4	3
**Drehimpuls**	0.77 [0.00–0.91]	4	20.71 [3.16–129.65]	4	0.85 [0.02–1.00]	3	4
**SK**	0.57 [0.00–0.83]	5	32.32 [5.48–128.51]	6	0.77 [0.09–0.98]	5	5
**AGHSSO**	0.48 [0.00–0.87]	6	24.11 [6.08–132.51]	5	0.64 [0.03–0.99]	7	6
**UNMC**	0.40 [0.00–0.76]	7	36.26 [11.58–116.97]	7	0.69 [0.07–0.91]	6	7
**SPPIN_SCNU**	0.24 [0.00–0.61]	8	93.54 [27.20–274.0]	9	0.58 [0.03–0.99]	8	8
**GIBI230**	0.21 [0.00–0.89]	9	63.41 [5.48–170.38]	8	0.31 [0.00–0.96]	9	9

**Table 5:** **The mean scores for the diagnostic and post-chemotherapy scans for each team, using Kruskal–Wallis tests.** Significant *p*-values (<0.05) are depicted in bold. The *p*-values of NaN resulted from NaNs in HD scores.

Team	Mean Dice Diagnostic	Mean Dice Post-Chemo	*p*-Value	Mean HD Diagnostic	Mean HD Post-Chemo	*p*-Value	Mean VS Diagnostic	Mean VS Post-Chemo	*p*-Value
**Blackbean**	0.89	0.63	0.01	6.40	30.17	0.69	0.94	0.75	0.05
**Jishenyu**	0.89	0.57	0.00	5.24	42.32	0.11	0.93	0.66	0.02
**Ouradiology**	0.80	0.51	0.03	11.51	39.31	0.20	0.83	0.61	0.11
**Drehimpuls**	0.76	0.50	0.07	12.55	48.04	0.40	0.79	0.62	0.24
**SK**	0.65	0.40	0.12	19.26	50.87	0.46	0.75	0.65	0.55
**AGHSSO**	0.66	0.39	0.07	31.50	54.11	0.08	0.79	0.53	0.08
**UNMC**	0.57	0.32	0.08	31.15	54.82	0.08	0.68	0.57	0.73
**SPPIN_SCNU**	0.35	0.19	0.15	53.43	118.93	0.01	0.51	0.55	1.00
**GIBI230**	0.66	0.24	0.03	31.31	83.64	NaN	0.75	0.33	0.05

## Data Availability

The data presented in this study are available on request from the corresponding author due to the sensitive nature of the data.

## Supplementary Materials

The following supporting information can be downloaded at: https://www.mdpi.com/article/10.3390/bioengineering12111157/s1.

## Abbreviations

The following abbreviations are used in this manuscript:

DCOG	Dutch Childhood Oncology Group
Dice score	Dice similarity coefficient
DL	Deep learning
DWI	Diffusion-weighted imaging
HD95	95th percentile Hausdorff distance
MICCAI	Medical Image Computing and Computer Assisted Intervention
MRI	Magnetic resonance imaging
nnU-Net	No-New-U-Net (self-adapting U-Net segmentation framework)
SPPIN	Surgical Planning in PedIatric Neuroblastoma
STU-Net	Scalable and Transferable U-Net
T1	T1-weighted MRI sequence
T2	T2-weighted MRI sequence
VS	Volumetric similarity
