# Supplementary material for: A Community Benchmark for the Automated Segmentation of Pediatric Neuroblastoma on Multi-Modal MRI: Design and Results of the SPPIN Challenge at MICCAI 2023
Source: Bioengineering (Basel). 2025 Oct 26;12(11):1157. doi: 10.3390/bioengineering12111157 (PMC12649702; doi:10.3390/bioengineering12111157)
Supplement: Supplementary file 1 [file bioengineering-12-01157-s001.zip › Supplementary File A.pdf]

Supplementary File A

1. Open the T1 scan in Mevislab.
2. Familiarize yourself with the tumor and surrounding structures.
3. Identify the most cranial slice in which you see neuroblastoma present. Segment this slice first.
4. Identify the most caudal slice, and segment it.
5. Segment all slices in between. If in doubt about tumor boarders, it might help to look at previous scanning moments or the other sequences of the patient (if available). Please note that the segmentation of the neuroblastoma is focused on the outline of the tumor. This means that you don't have to exclude the arteries and veins from this segmentation if fully encased by the tumor.
