# Supplementary material for: A Community Benchmark for the Automated Segmentation of Pediatric Neuroblastoma on Multi-Modal MRI: Design and Results of the SPPIN Challenge at MICCAI 2023
Source: Bioengineering (Basel). 2025 Oct 26;12(11):1157. doi: 10.3390/bioengineering12111157 (PMC12649702; doi:10.3390/bioengineering12111157)
Supplement: Supplementary file 1 [file bioengineering-12-01157-s001.zip › Supplementary File B.pdf]

Supplementary File B

Below, all methods of the participating teams are described in order of their ranking (from highest to lowest).

Blackbean: A Scalable and Transferable U-Net (STU-Net), which is based on nnU-Net, was pretrained on the large dataset TotalSegmentor (1204 CT images with 104 annotated structures) to create this teams segmentation method [24], [25], [31]. As input, this team used T_1-weighted contrast enhanced images only. Data augmentation during training included additive brightness, gamma, rotation, scaling, mirror and elastic deformation.

Jishenyu: The segmentation pipeline of this team started with registration of all sequences to the T_1-weighted scan. Next, they used a T_1-weighted contrast enhanced fill strategy for enhancing tumor visibility by replacing lower intensity pixels of the T_2-weighted and diffusion scan with higher intensity values from the corresponding T_1-weighted contrast enhanced scan. Data augmentation was used to enhance the dataset variability: Gaussian blur and noise, brightness multiplicate, contrast augmentation, simulation of low resolution, gamma transform, and mirror transform. The segmentation was performed using a pre-trained nnU-Net, for which details were not specified [25].

Ouradiology: This team proposed segmentation based on a nnU-Net, with as input only the T_1-weighted images [25]. After training with 5-fold cross validation, their final model was an ensemble of models as determined by nnU-Net.

Drehimpuls: After testing several different nnU-Net algorithms (including the addition of residual connections in the encoder), they showed no enhanced performance compared to a standard nnU-Net [25]. As loss, the F_β was used instead of the standard Dice loss [32]. A fallback network was fine-tuned on all scans that had a Dice score of < 0.5 during 5-fold cross validation. This fallback network was used as a postprocessing step in case the first network predicted < 1000 foreground pixels.

SK: A 2.5D convolutional neural net (CNN), using consecutive slices in a transverse plane, with EfficientNet as encoder was used by this team [33]. After optimalisation, they found that using 5 slices was the best performing depth for the 2.5D CNN. They used T_1- contrast enhanced and T_2-weighted images, which were resampled but not registered.

AGHSSO: Heavy data augmentation and the use of all four input sequences was central to this team's segmentation method. Using affine transformations, random intensity transformation, Gaussian noise, flipping along all axes, motion artifacts, anisotropy transformation, Gaussian blurring, and offline elastic transformations, they created a total of 10000 training cases. They used a 3D Res-Net as architecture, trained with a

combination of Soft Dice Loss and Focal Loss. In postprocessing, all connected components of 20 voxels or less were removed.

UNMC: This team used all four input sequences for their method, registered by a "QuickRigid" registration to the T_1-weighted contrast enhanced image [34]. The DynUNet model from MONAI was used as segmentation method [35]. Before training, they performed a background cropping (voxels with an intensity below the 90th percentile). Image normalization and resampling was also performed. During training random flips, rotations, scaling, and shifting of intensity values were used as augmentation. As postprocessing, the largest connected component of the prediction was selected.

SPPIN_SCNU: The method of this team only used T_1-weighted contrast enhanced images as input. Their method was based on a U-Net with a transformer as encoder, called UNETR [36].

GIBI230: A previously developed neuroblastoma segmentation method, developed as part of the PRIMAGE project, was used by this team [10], [17]. This is the only team that used the T_2-images as input. This team used their previously developed method on our dataset, without any finetune training specifically for our dataset.
